# Isolation and Identification of Lignans and Other Phenolic Constituents from the Stem Bark of *Albizia julibrissin* Durazz and Evaluation of Their Nitric Oxide Inhibitory Activity

**DOI:** 10.3390/molecules25092065

**Published:** 2020-04-28

**Authors:** Wei Li, Hye Jin Yang

**Affiliations:** Korean Medicine (KM) Application Center, Korea Institute of Oriental Medicine, Daegu 41062, Korea; hjyang@kiom.re.kr

**Keywords:** *Albizia julibrissin* Durazz, Fabaceae, lignan, phenolic, nitric oxide

## Abstract

A dibenzylbutane-type lignan (**16**), along with eight furofuran-type (**1**–**8**), five furan-type (**9**–**13**), two dibenzylbutane-type (**14** and **15**), two bibenztetrahydronaphthalene-type lignans (**17** and **18**), two neolignans (**19** and **20**), and six phenolic derivatives (**21**–**26**) were isolated from an MeOH extract of the stem bark of *Albizia julibrissin* Durazz. The chemical structures of the obtained compounds were elucidated by nuclear magnetic resonance (NMR) and mass spectrometry (MS) analyses. Of the evaluated compounds, 14 were isolated from *A. julibrissin* and the Fabaceae family for the first time. Anti-inflammatory effects of the isolated analogs were investigated in terms of the inhibition of the nitric oxide (NO) production in lipopolysaccharide (LPS)-stimulated murine RAW264.7 macrophage cells. Ten compounds (**10**–**12**, **14**, and **17**–**22**) displayed significant dose-dependent inhibitory effects against the NO production, with IC_50_ values ranging from 5.4 to 19.2 µM. Moreover, eight compounds (**1**–**4**, **9**, **13**, **15**, and **16**) exhibited moderate inhibitory activities, with IC_50_ values ranging from 21.0 to 62.5 µM.

## 1. Introduction

*Albizzia julibrissin* Durazz, commonly known as the mimosa or silk tree, belongs to a traditional Chinese medicine practice called He Huan, and is widely distributed in Korea, China, India, and other Asian regions. The dried stem bark of *A. julibrissin* has been utilized in traditional Chinese medicine to treat insomnia, melancholia, diuresis, asthenia, ascariasis, depression, anxiety, and confusion [1,2,3]. Previous pharmacological studies of *A. julibrissin* revealed its sedative, antidepressant, antitumor, anti-infertility, immunomodulatory, and antioxidant properties [4,5,6]. Furthermore, the bark extract can be applied to bruises, ulcers, abscesses, burns, hemorrhoids, and fractures, and has been shown to exhibit cytotoxic activity [7]. A previous study involving phytochemical analyses of *A. julibrissin* revealed that oleanane-type triterpenoid saponins were the principle constituents of this species. Compounds of this type feature acacic acid, aglycone, and several saccharide moieties at the C-3, C-21, and C-28 positions, respectively [8]. The presence of flavonoids, alkaloids, ceramides, phenolic glycosides, and steroids in the bark and leaves of this tree has also been reported [9,10,11,12]. *A. julibrissin* is recorded in the Chinese Pharmacopoeia as a sedative and anti-inflammatory agent used to treat injuries resulting from falls, and to remove carbuncles [13]. Nevertheless, lignans, phenolic compounds, and their associated bioactivities have not been sufficiently evaluated. Little is known about the presence of phenolic glycosides and lignans in this plant, and their respective biological effects have not been studied. In this study, we aimed to isolate new anti-inflammatory compounds which would complement the panel of known active components of *A. julibrissin*. We successfully isolated and elucidated the structure of a novel dibenzylbutane-type lignan, along with eight furofuran-, five furan-, two dibenzylbutane-, and two bibenztetrahydronaphthalene-type lignans, as well as two neolignans and six phenolic derivatives from an MeOH extract of the stem bark of *A. julibrissin*. Moreover, the inhibitory activity of the isolated compounds against nitric oxide (NO) production in lipopolysaccharide (LPS)-stimulated murine RAW264.7 macrophage cells and the structure–activity relationship were evaluated through utilizing various analytical techniques, including nuclear magnetic resonance (NMR) spectroscopy and mass spectrometry (MS).

## 2. Results and Discussion

### 2.1. Isolation and Structural Elucidation

**Figure 1:**
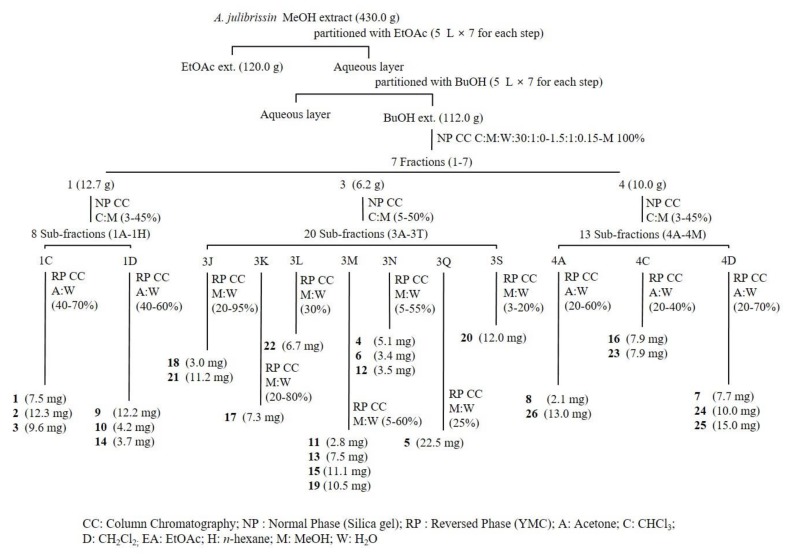
Schematic diagram displaying the process of isolation of compounds **1**–**26** from *Albizzia julibrissin*.

**Figure 2:**
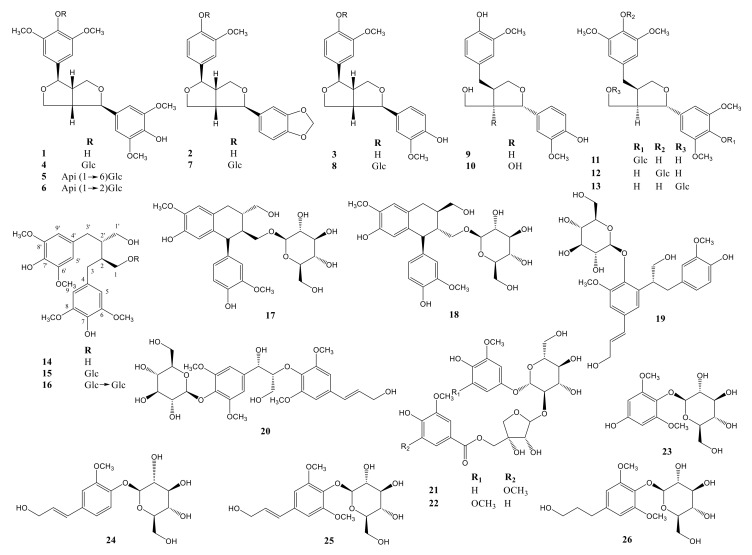
Structures of compounds **1**–**26** from *A. julibrissin.*

Using combined chromatographic separation techniques, 26 compounds (**1**–**26**), including eight furofuran-type (FF) lignans (**1**–**8**), five furan-type (FR) lignans (**9**–**13**), three dibenzylbutane-type (DB) lignans (**14**–**16**), two bibenztetrahydronaphthalene-type (BN) lignans (**17** and **18**), two neolignans (**19** and **20**), and six phenolic derivatives (**21**–**26**), were effectively isolated from an MeOH extract of *A. julibrissin* stem bark (Figure 1). The high-performance liquid chromatography (HPLC) evaluation showed that the purity of the isolated compounds was >90%. Based on the conducted analysis, the structures were determined as: (−)-lirioresinol B (**1**) [14], (−)-piperitol (**2**) [15], (−)-pinoresinol (**3**) [16], (−)-syringaresinol-4-*O*-β-d-glucopyranoside (**4**) [17], obtusifoside A (**5**) [18], (−)-syringaresinol-4-*O*-β-d-apiofuranosyl-(1→2)-β-d glucopyranoside (**6**) [14], simplexoside (**7**) [19], (−)-pinoresinol-β-d-glucopyranoside (**8**) [20], (+)-lariciresinol (**9**) [21], vladinol B (**10**) [21], (8*R*, 7’*S*, 8’*R*)-5,5’-dimethoxylariciresinol 4’-*O*-β-d-glucopyranoside (**11**) [22], manglieside E (**12**) [23], alangilignoside C (**13**) [19], (+)-(8*S*, 8’*S*)-bisdihydrosyringenin (**14**) [24], secoisolariciresinol (**15**) [25], julibrissinoside (**16**), (+)-(8*S*, 7′*S*, 8′*S*)-burselignan-9′-*O*-β-d-glucopyranoside (**17**) [26], (+)-(8*R*, 7′*S*, 8′*R*)-isolariciresinol-9′-O-β-d-fucopyranoside (**18**) [26], icariside E_5_ (**19**) [27], (7*S*,8*R*)-picraquassioside C (**20**) [28], albibrissinoside B (**21**) [29], khaephuoside B (**22**) [30], leonuriside A (**23**) [31], coniferin (**24**) [31], syringin (**25**) [31], and dihydrosyringin (**26**) [20]. The structures were confirmed by comparing the obtained spectroscopic data with the previously reported values (Figure 2). Among them, julibrissinoside (**16**) is a new compound. Moreover, 14 compounds (**2**, **5**, **7**, **11**–**18**, and **20**–**22**) were isolated from *A. julibrissin* and the Fabaceae family for the first time. To the best of our knowledge, the present study involved the first comprehensive chemical assessment of lignans and phenolic compounds from the *A. julibrissin* stem bark.

Compound **16** was obtained as a brown amorphous powder. The molecular formula was established as C_34_H_50_O_18_ by high-resolution electrospray ionization time-of-flight mass spectrometry (HR-ESI-TOF-MS; *m/z* 769.2890 ([M+Na]^+^)). The ^1^H-NMR spectrum of **16** (Table 1) exhibited four typical peaks corresponding to the 1,2,3,5-tetra-subsituted aromatic protons at δ_H_ 6.34 (s, H-5’/9’) and 6.40 ppm (s, H-5/9), and to four methoxy moieties at δ_H_ 3.72 (s, 6/8-OMe) and 6.40 ppm (s, 6’/8’-OMe). Furthermore, the presence of two peaks for anomeric protons at δ_H_ 4.28 (d, *J* = 8.0 Hz, H-1’’’) and 4.72 ppm (d, *J* = 7.8 Hz, H-1’’) indicated that **16** contains two sugar moieties. Notably, the larger coupling constants of the anomeric protons suggested the β-configuration of the two glucosyl scaffolds. Enzymatic hydrolysis of **16** yielded aglycone and glucose. The comparison with an authentic sample provided evidence for the identification of aglycone as (+)-(8*S*,8’*S*)-bisdihydrosyringenin (compound **14**) [24]. The absolute configurations of the two D-glucosyl moieties were established by gas chromatography (GC) analysis.

Accordingly, the obtained ^13^C-NMR spectrum (Table 1) contained eight signals corresponding to the quaternary carbon atoms of the aromatic rings at δ_C_ 130.0 (C-7), 130.1 (C-7′), 132.0 (C-4′), 132.5 (C-4), 148.9 (C-6′/8′), and 149.3 ppm (C-6/8), along with four aromatic carbon signals at δ_C_ 107.7 (C-5′/9′) and 107.8 ppm (C-5/9), and six carbon signals at δ_C_ 36.1 (C-3), 36.3 (C-3′), 41.3 (C-2), 44.0 (C-2′), 63.1 (C-1′), and 70.8 ppm (C-1), which were consistent with compound **14 [24]**. The structure of the skeleton was identified by elucidation of the observed heteronuclear multiple bond correlations (HMBCs) at δ_H_ 2.14 (H-2′)/δ_C_ 132.0 (C-4′) and δ_H_ 2.35 (H-2)/δ_C_ 132.5 (C-4). The noted HMBC between C-1 (δ_H_ 3.61/4.06 and δ_C_ 70.8) and C-1′′ (δ_H_ 4.72 and δ_C_ 105.4) implied the presence of a glucosyl moiety attached at the C-1 position of the main skeleton. It is noteworthy that this observation was previously reported for compound **15** (secoisolariciresinol) [25]. The difference between the two compounds was that **16** contained a glucosyl group at the C-6′′ position. This finding was evidenced by the observation of a key HMBC between H-1′′′ (δ_H_ 4.28) and C-6′′ (δ_C_ 70.8). Based on these data, the structure of **16** was elucidated and named julibrissinoside.

### 2.2. Bioassays

To evaluate the anti-inflammatory activities of the isolated compounds **1**–**26**, we assessed the LPS-induced production of inflammatory mediators such as NO by RAW 264.7 cells. Cell viability was measured utilizing the 3-(dimethylthiazol-2-yl)-2,5-diphenyltetrazolium bromide (MTT) assay. The results demonstrated that none of the compounds affected cell viability (100 μM, data not shown). Furthermore, NO production was measured by employing Griess reaction assays. The obtained outcomes showed that 18 compounds exhibited significant inhibitory activities against NO production. Of them, 10 compounds (**10**–**12**, **14**, and **17**–**22**) showed strong dose-dependent inhibitory effects (IC_50_ < 20 µM), with IC_50_ values of 6.5 ± 0.1, 18.3 ± 0.3, 19.2 ± 0.3, 11.8 ± 0.2, 10.1 ± 0.2, 12.3 ± 0.7, 5.4 ± 0.1, 7.7 ± 0.2, 8.9 ± 0.3, and 8.3 ± 0.1 µM, respectively (Table 2). Compounds **1**–**4**, **9**, **13**, **15**, and **16** exhibited moderate inhibitory activities (20 µM < IC_50_ < 100 µM), with IC_50_ values ranging from 21.0 to 62.5 µM, while compounds **5**–**8** and **23**–**26** showed weaker inhibitory effects (IC_50_ > 100 µM).

Upon investigating the structure–activity relationship of the isolated compounds, it was determined that, among the furofuran-type lignans (**1**–**8**), compounds **1** and **3** displayed the strongest inhibitory effects. This outcome indicated that the number of substituents on the aromatic rings and sugar units were important factors for the observed inhibitory activity. When the sugar units were linked to the aglycone, the effects weakened; when the methoxy group was linked to C-3 and C-5, the anti-inflammatory activity increased significantly. Moreover, all the furan-type lignans (**9**–**13**) displayed strong activities. Particularly strong activity was determined for compound **10**, which contains a hydroxyl moiety at the C-2 position that appears to play a role in the NO inhibitory activity. On the other hand, the inhibitory effects of dibenzylbutane- and bibenztetrahydronaphthalene-type lignans (**14**–**18**), which exhibit similar structures, are believed to be dependent on the number of sugar moieties. Previous studies reported that some lignans inhibit NO production [32,33,34]. However, the structure–activity relationship did not provide elaborate detail, especially on the substituents of aromatic rings. These results imply that the numbers of sugar units, substituents on the aromatic rings, and hydroxyl groups are significant in the process of inhibiting NO production.

## 3. Conclusions

In this study, 26 compounds were successfully isolated from *A. julibrissin*. Julibrissinoside (**16**), a novel compound, as well as 25 additional known natural products were isolated. To the best of our knowledge, the current work comprises the first comprehensive report on lignans, the phenolic components of *A. julibrissin*, as well as their associated NO production inhibitory activity. These obtained results confirm that both lignans and phenolic compounds constitute new bioactive components of *A. julibrissin*. Overall, the outcomes of our study provide a valuable platform for the application of lignans and phenolic analogs isolated from *A. julibrissin* in the treatment of inflammatory diseases.

## 4. Materials and Methods

### 4.1. General Information

Optical rotations were determined using a Jasco DIP-370 automatic polarimeter. The NMR spectra were recorded using a JEOL ECA 600 spectrometer (^1^H, 600 MHz; ^13^C, 150 MHz). The LCQ advantage trap mass spectrometer (Thermo Finnigan, San Jose, CA, U.S.A.) was equipped with an electrospray ionization (ESI) source, and high-resolution electrospray ionization mass spectra (HR-ESI-MS) were obtained using an Agilent 6530 Accurate-Mass Q-TOF LC/MS system. Column chromatography was performed using a silica gel (Kieselgel 60, 70-230, and 230-400 mesh, Merck, Darmstadt, Germany), YMC RP-18 resins, and thin layer chromatography (TLC) was performed using pre-coated silica-gel 60 F_254_ and RP-18 F_254_S plates (both 0.25 mm, Merck, Darmstadt, Germany).

### 4.2. Plant Material

Dried stem bark of *A. julibrissin* was provided by Bomyeong Herbal Market, Seoul in 2017, and taxonomically identified by Dr. Wei Li. A voucher specimen (KM-004101) was deposited at Korean Medicine (KM) Application Center, Korea Institute of Oriental Medicine, Republic of Korea.

### 4.3. Extraction and Isolation

The dried stem bark of *A. julibrissin* (3.0 kg) was extracted with MeOH (5 L × 3 times) under reflux condition. The solvents were evaporated using a rotary vacuum evaporator to provide MeOH extract (150.0 g). The MeOH extract was suspended in water and partitioned with EtOAc and *n*-BuOH. The BuOH fraction (112.0 g) was subjected to silica gel (10 × 30 cm) column chromatography with CHCl_3_-MeOH-H_2_O (10:1:0–1.5:1:0.15–MeOH 100%) to give seven fractions (Fr. 1–7). Fraction 1 (12.7 g) was subjected to silica gel (2.5 × 80 cm) column chromatography with CHCl_3_-MeOH (MeOH 3–45%) elution solvent to give 8 sub-fractions (Fr. 1A–1H). Fraction 1C was separated using YMC (1 × 80 cm) column chromatography with an acetone-H_2_O (acetone 40–70%) elution solvent to give (−)-lirioresinol B (1) (7.5 mg), (−)-piperitol (2) (12.3 mg), and (−)-pinoresinol (3) (9.6 mg). Fraction 1D was separated using YMC (1 × 80 cm) column chromatography with an acetone-H_2_O (acetone 40–60%) elution solvent to give (+)-lariciresinol (9) (12.2 mg), vladinol B (10) (4.2 mg), and (+)-(8*S*,8′*S*)-bisdihydrosyringenin (14) (3.7 mg).

Fraction 3 (6.2 g) was subjected to silica gel (2.0 × 80 cm) column chromatography with CHCl_3_-MeOH (MeOH 5–50%) elution solvent to give 20 sub-fractions (Fr. 3A–3T). Fraction 3J was separated using YMC (1.0 × 80 cm) column chromatography with an MeOH-H_2_O (MeOH 20–95%) elution solvent to give (+)-(8*R*,7′*S*,8′*R*)-isolariciresinol-9′-*O*-β-d-fucopyranoside (18) (3.0 mg) and albibrissinoside B (21) (11.2 mg). Fraction 3K was separated using YMC (1.0 × 80 cm) column chromatography with an MeOH-H_2_O (MeOH 20–80%) elution solvent to give (+)-(8*S*,7′*S*, 8′*S*)-burselignan-9′-O-β-D-glucopyranoside (17) (7.3 mg). Fraction 3L was separated using YMC (1.0 × 80 cm) column chromatography with an MeOH-H_2_O (MeOH 30%) elution solvent to give khaephuoside B (22) (6.7 mg). Fraction 3M was separated using YMC (1.0 × 80 cm) column chromatography with an MeOH-H_2_O (MeOH 5–60%) elution solvent to give (8*R*,7**′***S*,8**′***R*)-5,5**′**-dimethoxylariciresinol 4**′**-*O*-β-d-glucopyranoside (11) (2.8 mg), alangilignoside C (13) (7.5 mg), secoisolariciresinol (15) (11.1 mg), and icariside E_5_ (19) (10.5 mg). Fraction 3N was separated using YMC (1.0 × 80 cm) column chromatography with an MeOH-H_2_O (MeOH 5–55%) elution solvent to give (−)-syringaresinol-4-*O*-β-d-glucopyranoside (4) (5.1 mg), (−)-syringaresinol-4-*O*-β-d-apiofuranosyl-(1→2)-β-d glucopyranoside (6) (7.5 mg), and manglieside E (12) (3.5 mg). Fraction 3Q was separated using YMC (1.0 × 80 cm) column chromatography with an MeOH-H_2_O (MeOH 25%) elution solvent to give obtusifoside A (5) (22.5 mg). Fraction 3S was separated using YMC (1.0 × 80 cm) column chromatography with an MeOH-H_2_O (MeOH 3–20%) elution solvent to give (7*S*,8*R*)-picraquassioside C (20) (12.0 mg).

Fraction 4 (10.0 g) was subjected to silica gel (3.0 × 80 cm) column chromatography with CHCl_3_-MeOH (MeOH 3–45%) elution solvent to give 13 sub-fractions (Fr. 4A–4M). Fraction 4A was separated using YMC (1.0 × 80 cm) column chromatography with an acetone-H_2_O (acetone 20–60%) elution solvent to give (−)-pinoresinol β-D-glucopyranoside (8) (2.1 mg) and dihydrosyringin (26) (13.0 mg). Fraction 4C was separated using YMC (1.0 × 80 cm) column chromatography with an acetone-H_2_O (acetone 20–40%) elution solvent to give julibrissinoside (16) (7.9 mg) and leonuriside A (23) (7.9 mg). Fraction 4D was separated using YMC (1.0 × 80 cm) column chromatography with an acetone-H_2_O (acetone 20–70%) elution solvent to give simplexoside (7) (7.7 mg), coniferin (24) (10.0 mg), and syringin (25) (15.0 mg).

Julibrissinoside (**16**): brown amorphous powder; ${\left[\alpha \right]}_{D}^{25}$: –38.3 (*c* 0.1, MeOH); ^1^H NMR (methanol-*d*_4_, 600 MHz) and ^13^C NMR data (methanol-*d*_4_, 150 MHz), see Table 1; HR-ESI-MS: *m/z* 769.2890 [M + Na]^+^ (calcd. for 769.2890).

### 4.4. Enzymatic Hydrolysis

Compound **16** (3.0 mg) was mixed with β-glucosidase (3.0 mg) in water (1.0 mL) and the solution was shaken in a water bath at 37 °C for 12 h. Subsequently, the reaction mixture was concentrated and subjected to silica gel column chromatography (1.0 × 15.0 cm, 40–63 μm) using CHCl_3_-MeOH (15:1, 60 mL) and CHCl_3_-MeOH-H_2_O (7:3:0.5, 60 mL) as the solvent systems to afford aglycone **16a** (1.2 mg) and a sugar fraction. The sugar fraction was concentrated until dried using N_2_ gas. The resulting residue was dissolved in anhydrous pyridine (0.1 mL), and L-cysteine methyl ester hydrochloride in pyridine (0.06 M, 0.1 mL) was then added to the solution. Following heating the reaction mixture at 60 °C for 2 h, 0.1 mL of trimethylsilylimidazole was added. Heating at 60 °C was continued for another 1.5 h. The dried product was partitioned with *n*-hexane and H_2_O (0.1 mL each), and the organic layer was analyzed using gas chromatography (GC): DB-5 capillary column (0.32 mm × 30 m); FID detector; column temp., 210 °C; injector temp., 270 °C; detector temp., 300 °C; carrier gas, He (2 mL/min). Under these conditions, the standard sugars gave peaks at *t*R (min) = 14.12 and 12.24 for L- and D-glucose, respectively. The peak of the hydrolysate of **16** was detected at tR (min) = 12.21, which was identified as D-glucose by comparison with the retention time of the authentic samples following treatment with trimethylsilylimidazole in pyridine.

### 4.5. Cell Culture and Stimulation

The RAW 264.7 cells were obtained from the Korean Cell Line Bank (KCLB, Chongno-gu, Seoul, Korea) and maintained in RPMI 1640 medium supplemented with 10% fetal bovine serum (FBS) and 100 U/mL penicillin at 37 °C in a humidified incubator containing 5% CO_2_. The cells were cultured at 2 × 10^5^ cells/mL in RPMI 1640 medium containing 10% FBS in 96-well tissue culture plates for 18 h and were subsequently pretreated with 0.4, 2, 10, 50, and 100 μM of the analyzed compounds 1 h prior to stimulation with LPS (1 μg/mL) for 24 h in an incubator.

### 4.6. Cell Viability

The cell viability was evaluated using the MTT method. Briefly, MTT was added to the cell culture medium for 4 h. The supernatant was then removed and the formazan crystals were dissolved in dimethyl sulfoxide (DMSO). The absorbance was measured at 540 nm. The percentage of dead cells was determined relative to the control group.

### 4.7. Nitric Oxide Assay

Nitrite, which accumulated in the culture medium, was measured using the Griess reaction as an indicator for the NO production. Briefly, the cell culture medium (100 μL, without phenol red) was mixed with an equal volume of the Griess reagent containing equal volumes of 1% (*w/v*) sulfanilamide in 5% (*v/v*) H_3_PO_4_ and 0.1% (*w/v*) naphthylethylenediamine-HCl. The solution was incubated at room temperature for 20 min, after which the absorbance was measured at 520 nm using a microplate reader. Fresh culture medium was employed as the blank in all experiments. The amounts of nitrite in the samples were established using the NaNO_2_ serial dilution standard curve.

### 4.8. Statistical Analysis

All data are represented as means ± SD of at least three independent experiments performed in triplicate. Statistical significance is indicated as determined by one-way ANOVA, followed by Dunnett’s multiple comparison test (*p* < 0.05) utilizing GraphPad Prism 6.0 (GraphPad Software Inc., San Diego, CA, USA).

## Figures and Tables

**Table 1:** ^1^H and ^13^C NMR spectroscopic data for compound **16** in methanol-*d_4_*.

Position	δ_H_^a^ (*J*/Hz)	δ_C_^b^	Position	δ_H_^a^ (*J*/Hz)	δ_C_^b^
1	3.61 m^c^ 4.06 dd (6.0, 9.4)	70.8	1′′	4.72 d (7.8)	105.4
2	2.35 m^c^	41.3	2′′	3.69–3.75 m	76.3
3	2.70 dd (14.0, 7.0) 2.82 dd (14.0, 7.0)	36.1	3′′	3.69–3.75 m	79.0
4		132.5	4′′	3.69–3.75 m	72.1
5	6.40 s	107.8	5′′	3.79–3.90 m	78.6
6		149.3	6′′	4.28 m 4.23 m	70.8
7		130.0	1′′′	4.28 d (8.0)	105.0
8		149.3	2′′′	3.79–3.90 m	75.6
9	6.40 s	107.8	3′′′	3.79–3.90 m	78.3
1′	3.69 m 3.90 dd (6.0, 11.0)	63.1	4′′′	3.69–3.75 m	71.8
2′	2.14 m	44.0	5′′′	3.79–3.90 m	78.8
3′	2.64 dd (11.0, 13.7) 2.81 dd (7.0, 13.7)	36.3	6′′′	3.50 m	62.0
4′		132.0	6-OMe	3.72 s	56.5
5′	6.34 s	107.7	8-OMe	3.72 s	56.5
6′		148.9	6′-OMe	3.82 s	56.7
7′		130.1	8′-OMe	3.82 s	56.7
8′		148.9			
9′	6.34 s	107.7			

Assignments were achieved by the analysis of the HMQC and HMBC experiments; *J* values (Hz) are given in parentheses. ^a^ 600 MHz. ^b^ 150 MHz. ^c^ Overlapped.

**Table 2:** NO inhibitory effects of isolated compounds **1**–**26.**

Compounds	IC_50_ (µM)	Compounds	IC_50_ (µM)
**1**	28.1 ± 0.8	**14**	11.8 ± 0.2
**2**	42.6 ± 1.1	**15**	38.7 ± 1.9
**3**	31.1 ± 0.3	**16**	62.5 ± 1.6
**4**	52.7 ± 1.2	**17**	10.1 ± 0.2
**5**	>100	**18**	12.3 ± 0.7
**6**	>100	**19**	5.4 ± 0.1
**7**	>100	**20**	7.7 ± 0.2
**8**	>100	**21**	8.9 ± 0.3
**9**	21.0 ± 1.5	**22**	8.3 ± 0.1
**10**	6.5 ± 0.1	**23**	>100
**11**	18.3 ± 0.3	**24**	>100
**12**	19.2 ± 0.3	**25**	>100
**13**	21.7 ± 1.8	**26**	>100
Quercetin^a^	15.6 ± 0.4		

Values represent means ± SD (*n* = 3). ^a^ Quercetin was used as a positive control.
